# Slow Cortical Potentials and Amplification—Part II: Acoustic Measures

**DOI:** 10.1155/2012/386542

**Published:** 2012-10-31

**Authors:** Lorienne M. Jenstad, Susan Marynewich, David R. Stapells

**Affiliations:** School of Audiology and Speech Sciences, The University of British Columbia, Vancouver, BC, Canada V6T 1Z3

## Abstract

In a previous study, we investigated slow cortical potential (SCP) N1-P2 amplitudes and N1 latencies in aided and unaided conditions, with the finding that despite being set to provide 20 or 40 dB of gain, none of the hearing aids resulted in a reliable increase in SCP response amplitude relative to the unaided (Marynewich et al., in press). The current study investigates the effects of hearing-aid processing on acoustic measures for two 1000-Hz tonal stimuli: short (60 ms) and long (757 ms), presented at three intensities (30, 50, 70 dB SPL) in aided and unaided conditions using three hearing aids (Analog, DigitalA, DigitalB) with two gain settings (20, 40 dB). Acoustic results indicate that gain achieved by the hearing aids, measured at 30 ms after stimulus onset, for both the short and long stimuli, was less than real-ear insertion gain measured with standard hearing aid test signals. Additionally, the digital hearing aids altered the rise time of the stimuli such that maximum gain was reached well past 30 ms after stimulus onset; rise times differed between the digital aids. These results indicate that aided SCP results must be cautiously interpreted and that further research is required for clinical application.

## 1. Introduction

Slow cortical potentials (SCPs) are being considered for their application in hearing aid fitting, particularly for infants [1–7]. SCP studies related to this purpose have produced mixed results; thus it is not known whether SCPs provide an accurate measure of the brain's response to (and, hopefully, behavioural perception of) signals processed by hearing aids, particularly hearing aids with digital signal processing. Several studies have shown that SCPs can be reliably recorded in aided conditions [2, 4–10], and some have reported that stimuli can be reliably differentiated in the SCP response in aided conditions [5, 6]. In contrast, there is a puzzling finding that the provision of gain via a hearing aid does not lead to the expected increase in SCP response amplitude for either tonal or speech stimuli [2, 6, 11]. To understand why SCP amplitude does not increase with hearing aid gain, it is important to quantify the acoustic effects of hearing-aid processing on the test signal; otherwise, the stimulus used to evoke the SCP in aided conditions is not known. This idea is supported by earlier studies which found that due to hearing-aid processing, stimuli used to measure some auditory-evoked potentials (AEP) may not result in valid measurements of hearing aid gain or output [12, 13]. 

Research on AEPs measured with hearing aids in place has yielded varying degrees of success. One reason for this variability may be due to the stimuli used to measure them. More specifically, although there are many commercially available test signals that may be used to assess hearing aids electroacoustically and/or via AEPs (including tonal and complex stimuli), not all stimuli are appropriate for measuring both hearing-aid processing and AEPs. For instance, complex stimuli such as speech-weighted composite noise provide better estimates of gain for speech compared with tonal stimuli [14–16], whereas tonal stimuli provide better measures of maximum power output (MPO) than do complex stimuli [15]. However, neither of these stimuli may be best for eliciting a given AEP, and the stimuli used to elicit an AEP may not be best for measuring hearing aids [12, 13, 17]. For example, research on hearing-aid processed stimuli has revealed that the click and brief-tone stimuli used in ABR testing are too short to activate the compression processing and steady-state response of the hearing aid [12, 13]. 

The N1 response does not reflect stimulus changes beyond the first 20–40 ms [18–22], and rise times between 20 and 30 ms result in the largest N1 amplitudes [20, 21]. The tonal stimulus used by Billings et al. [2] was atypical for SCP stimuli in that it had a more rapid rise time (7.5 ms) than is required to elicit a large-amplitude SCP and maintain reasonable stimulus frequency specificity; their stimulus was also much longer in duration than can be reflected by the SCP. The possibility that stimulus characteristics were the reason for the inability to measure hearing aid gain via SCPs was addressed by Marynewich et al. [11], who compared N1-P2 amplitudes and N1 latencies in unaided and aided conditions in normal-hearing listeners, using a stimulus designed to elicit larger N1 amplitudes with less compromise of frequency specificity; that is, a 60-ms duration tonal stimulus with a 20-ms rise time [20, 21, 23]. The results of Marynewich et al. [11] were similar to those of Billings et al. [2] in that the SCP amplitude did not increase as expected despite the provision of 20 or 40 dB of gain. 

What is not clear from many of the SCP studies is what effect hearing-aid processing had on the stimuli. Billings et al. [24] have since examined the effect of signal-to-noise ratio (SNR) and showed that the SNR may have been similar across aided and unaided conditions in their previous research [2, 6], which may explain why N1 amplitudes were not larger in the aided conditions compared with unaided. 

The purpose of this study was to measure, in the ear canals of subjects, tonal stimuli used for SCP testing before and after hearing-aid processing to determine how hearing-aid processing affected the stimuli, measured under the same conditions as the SCP testing. Of particular interest was whether there would be a difference between the gain measured with standard hearing aid test system stimuli and that measured with the stimuli used for the cortical measures. Also of interest was whether there would be a differential effect of analog and digital processing on gain, particularly within the first 30 ms after stimulus onset, even with all advanced features disabled. 

## 2. Materials and Methods

### 2.1. Subjects

Five subjects participated (mean age: 23 ± 2.1 years; 4 females). Four of these subjects also participated in Marynewich et al. [11]. Subjects were briefed on the study procedures and provided informed written consent prior to participating. All subjects were screened for normal middle/outer-ear function by immittance audiometry. Normal tympanograms were defined by a single-peak static admittance between ±50 daPa in response to a 226-Hz probe tone [25]. Although hearing status would not be expected to influence the results of the acoustic recordings, all subjects had normal hearing.

### 2.2. Hearing Aids

The same three behind-the-ear hearing aids, coupled with Comply snap tip 9-mm foam earmolds, were used for each participant: (i) Oticon E27 (“Analog”), (ii) Phonak Savia 211 dSZ (“DigitalA”), and (iii) Siemens Acuris S (“DigitalB”). These were the same hearing aids and settings used in our previous study [11]. 

The digital hearing aids were set, using NOAH 3 and the NOAHLink, to have two programs: one with 20 dB and the second with 40 dB real-ear insertion gain (REIG) for a 50 dB SPL 1000 Hz pure tone, as verified with the Fonix 7000 real-ear system. Both programs were set with a 1 : 1 compression ratio across the frequency range and were verified for linear processing using input/output coupler measures. All additional hearing aid features such as digital noise reduction and feedback management were disabled. Other frequencies were set to minimum gain. Settings for the digital instruments were saved in the NOAH 3 software for each subject and recalled in follow-up sessions. 

Gain settings for the analog hearing aid were achieved by setting the volume control to 1 (minimum) and turning the dB SPL trim-pot until the REIG was 20 dB for a 1000-Hz pure tone at a 50 dB SPL input level. To achieve the 40-dB gain setting, the volume control wheel was turned up until REIG equalled 40 dB at 1000 Hz. The volume control wheel was then marked for that setting. These gain settings were remeasured in follow-up sessions. 

REIG measures at 1000 Hz for all subjects and hearing aids are given in Table 1.

### 2.3. Stimuli

Two 1000-Hz “SCP” stimuli were used for the acoustic measures: (i) a stimulus of 60-ms total duration (including a 20-ms rise/fall time), the same as used by Marynewich et al. [11], and (ii) a stimulus of 757-ms duration (with a 7.57-ms rise/fall time), similar to the one used by Billings et al. [2]. Stimuli were presented with offset-to-onset interstimulus intervals (ISI) of 940 ms. Stimuli generated by Neuroscan's Stim2 software were further amplified by a Wavetek Rockland 852 filter (providing 20 dB of amplification below 3000 Hz), routed through a Tucker Davis Technologies (TDT) PA5 attenuator and HB7 headphone driver, and finally to a speaker in the sound field placed at 1.5 meters from the subject at 0° azimuth. The stimulus output at 80 dB SPL was calibrated with a Larson-Davis sound level meter by measuring the level of a longer-duration 1000-Hz tone (2-s duration, 20-ms rise/fall time; equal in peak-to-peak amplitude to the 60-ms 1000-Hz stimulus) at the head height of the subject, 1.5 m from the speaker. Stimuli were presented at three intensities (30, 50, and 70 dB SPL). 

### 2.4. Procedure

Subjects were asked to complete two test sessions, lasting no longer than three hours each and were given the choice of completing the sessions sequentially or on separate days. Procedures were approved by the University of British Columbia Behavioural Research Ethics Board. Subjects were screened for normal outer- and middle-ear function at each test session to ensure no changes across test sessions. 

Following hearing aid programming, all testing was conducted in a double-walled sound-attenuating booth. Average octave-band noise levels in the sound-attenuated booth at 0.5, 1, 2, and 4 kHz were 12, 10, 10, and 12 dB SPL, respectively. There were 36 test conditions (i.e., 18 for each of the short and long stimuli) and presentation order for each subject was randomly assigned prior to the test date(s). During testing, participants were asked to sit as still as possible while watching a movie of their choice in closed-captioning and no audio. Subjects sat in a reclining chair set in the upright position so that each participant was seated with their head above the chair back, the same position used for the previous SCP measurements. 

#### 2.4.1. Recording

An ER7C probe-tube output (set to provide 20 dB of attenuation) was routed through a second (passive) attenuator to Channel 1 of the Neuroscan recording system. The second attenuator ensured that input was not clipped by the recording system. The recording channel was amplified, filtered (0.05–3500 Hz), and digitized (20,000 Hz) by the Neuroscan Synamps^2^ system and averaged and analyzed by the Neuroscan Scan analysis system, using a 204.75-ms analysis time for the short stimulus (including a 70-ms prestimulus baseline) and a 960-ms analysis time for the long stimulus (including a 100-ms prestimulus baseline). The stimulus was recorded in the ear canal for each test condition until at least 100 accepted trials were obtained. Single-trial epochs were saved for offline processing, which included baseline correction across the stimulus duration and averaging of the single trials.

### 2.5. Data Analysis

Acoustic measures of interest were (i) gain at 30 ms after stimulus onset, (ii) maximum gain, and (iii) latency of maximum gain, or “rise time” (defined as the time at which the amplitude first reached 90% of maximum amplitude relative to an individually determined 0-ms point). Actual gain values were calculated for 20- and 40-dB hearing-aid gain conditions by determining the relative amplitude differences between aided and unaided stimulus waveforms from averaged recordings in the ear canal. A measurement point of 30 ms after stimulus onset was chosen because several studies indicate this is the most effective rise time and evokes the largest N1-P2 amplitudes; increases in stimulus levels beyond 20–40 ms have little to no effect on SCP amplitudes [18–22]. Maximum gain was calculated to determine the maximum gain produced at any time during the stimulus, even if this occurred past 30 ms after stimulus onset. Rise time was measured in order to determine whether hearing-aid processing resulted in stimulus rise times longer than 30 ms. The stimulus onsets (0-ms points) were determined for each waveform by a research assistant blind to the study purpose. Using the same zoom settings to visually inspect each waveform, the research assistant identified the time point at which there was periodicity in the recording. A random subset of the waveforms were retested to determine test-retest reliability of the 0-point identification protocol; the average of the absolute values of the errors was less than 1 ms (0.24 ms). 

### 2.6. Statistical Analysis

For the short-duration (60-ms) stimulus, two repeated-measures analyses of variance (ANOVA) were conducted for each of the dependent variables: gain measured at 30 ms, maximum gain, and rise time: (i) to measure the effects of the 20-dB gain setting, a two-way repeated-measures ANOVA was conducted comparing three levels of hearing aid type (Analog, DigitalA, and DigitalB) and three input levels (30, 50, and 70 dB SPL) and (ii) to measure the effects of the 40-dB gain setting, a two-way repeated-measures ANOVA was conducted comparing three levels of hearing aid type (Analog, DigitalA, and DigitalB) and two input levels (30 and 50 dB SPL). The same four repeated-measures ANOVAs were performed for the long duration (757-ms) stimulus.

Due to the exploratory nature of this study, main effects and interactions for all analyses were considered significant if *p* < .10. Huyn-Feldt correction factors were applied to the degrees of freedom and reported where appropriate (i.e., when the assumption of sphericity was not met). Significant interactions were examined by analyzing the simple main effects, then conducting paired *t*-tests for any significant simple main effects. Neuman-Keuls post hoc analyses were performed for significant main effects not involved in an interaction. Post hoc analyses were considered statistically significant if *p* < .10.

## 3. Results

The following section is divided into: (i) gain and rise time results for the short stimulus and (ii) gain and rise time results for the long stimulus. Mean data for gain measured at 30 ms and maximum amplitude, along with the rise times for maximum amplitude, are provided in Table 2. ANOVA results are reported in Tables 3 and 4, along with the results for Simple Main Effects when an interaction was significant.

### 3.1. Short Stimulus

**Figure 1:**
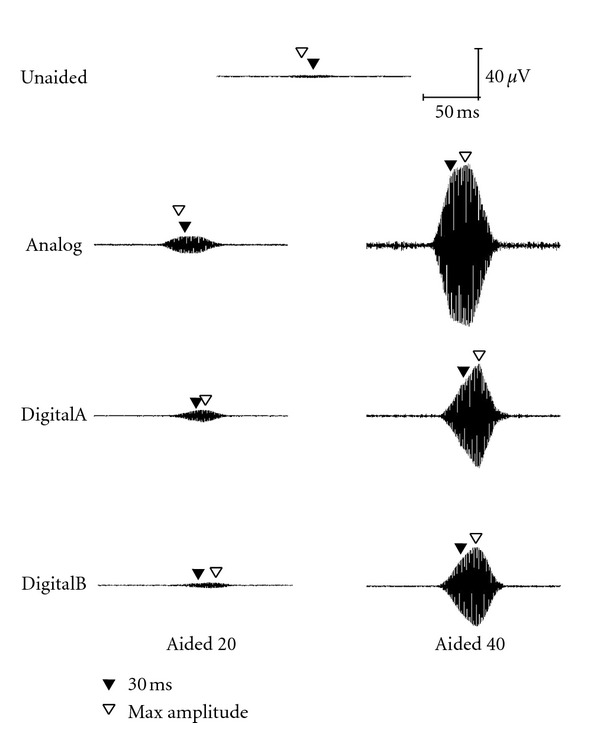
Waveform of the short stimulus presented at 30 dB SPL as measured in the ear canal of a single representative subject (subject 2), where “Aided 20” indicates the 20-dB gain condition and “Aided 40” indicates the 40-dB gain condition. The closed triangle indicates the point 30 ms after stimulus onset and the open triangle indicates the point at which maximum amplitude was reached.

**Figure 2:**
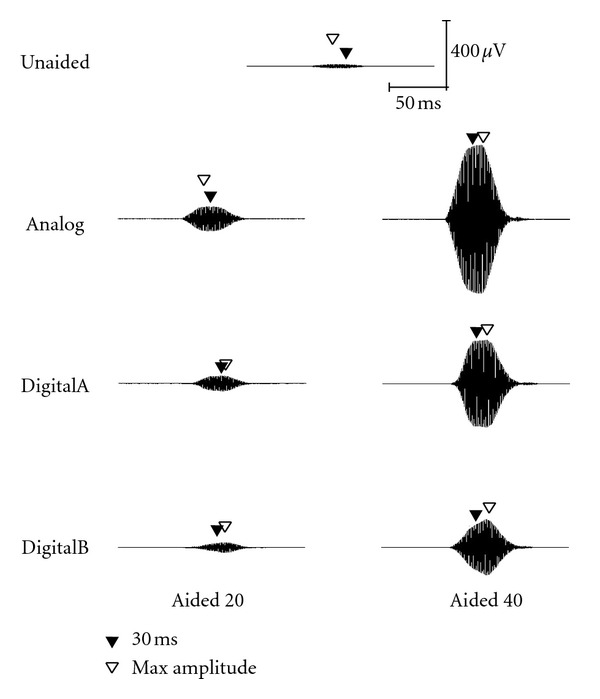
Waveform of the short stimulus presented at 50 dB SPL as measured in the ear canal of a single representative subject (subject 2).

**Figure 3:**
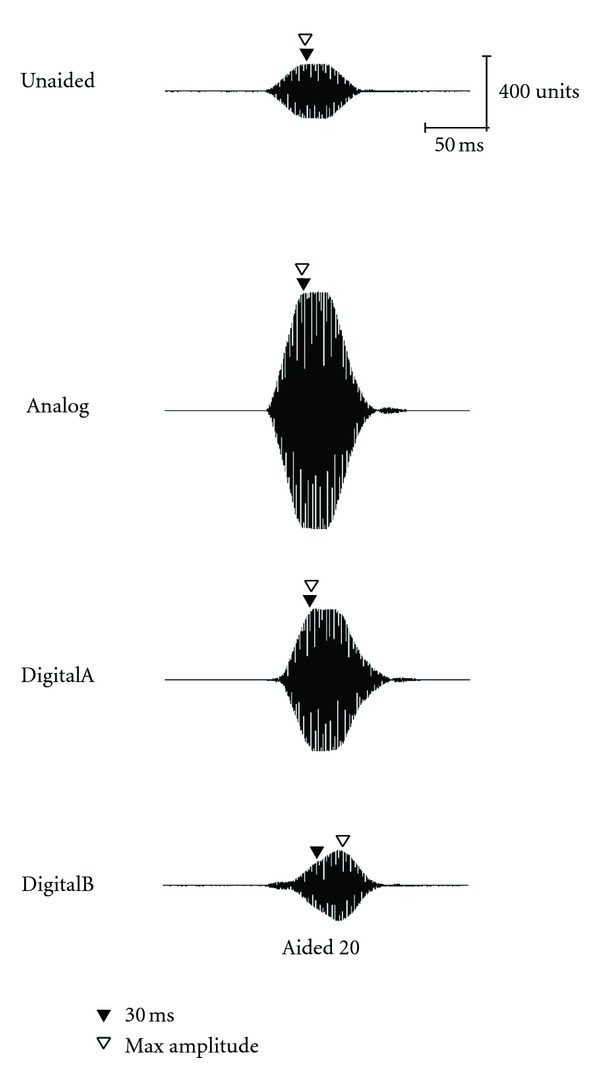
Waveform of the short stimulus presented at 70 dB SPL as measured in the ear canal of a single representative subject (subject 2).

Acoustic waveforms for the short stimulus (60-ms duration) in both unaided and aided conditions (Analog, DigitalA, and DigitalB) are presented for 30, 50, and 70 dB SPL input levels in Figures 1, 2, and 3, respectively. Both 20- and 40-dB gain settings are depicted where appropriate (e.g., 30 and 50 dB SPL input levels) and 30-ms and maximum amplitude measurement points are depicted by closed and open triangles, respectively. All figures in the following section illustrate the acoustic measures for a single subject representative of the overall pattern (subject no. 2). Note that for optimum visual representation, the scale is different across stimulus waveform figures. 

#### 3.1.1. 20-dB Gain Condition: Short Stimulus—Gain at 30 ms after Stimulus Onset 

Mean gain values presented in Table 2 indicate that at the 30-ms measurement point for the short stimulus, all hearing aids provided less than 20 dB gain. The analog hearing aid was 3-4 dB below the nominal gain across input levels; DigitalA was 4–9 dB below nominal gain, and DigitalB provided no measurable gain at 30 ms, and even attenuation of up to 3 dB.

Effect of Input Level: results from the ANOVA and post hoc analysis revealed that even though all three hearing aids were set to provide linear amplification, less gain was measured for the 30 dB SPL compared with the 50 dB SPL input level for all three hearing aids (*p* < .1) and the 30 dB SPL compared with the 70 dB SPL input level for all three hearing aids (*p* < .1). Gain was not significantly different between the 50 and 70 dB SPL input levels for any of the hearing aids (*p* > .1). 

Effect of Hearing Aid: the DigitalB hearing aid provided significantly less gain than both the DigitalA and Analog hearing aids at every input level (*p* < .01). The DigitalA hearing aid provided significantly less gain than the Analog hearing aid for the 30 dB SPL input (*p* < .05). There was no significant difference between gain provided by Analog and DigitalA aids for the higher input levels, 50 and 70 dB SPL (*p* > .1). 

#### 3.1.2. 20-dB Gain Condition: Short Stimulus—Gain at Maximum Amplitude 

Mean gain values presented in Table 2 show that, when measured at the maximum amplitude measurement point, once again, all of the hearing aids provided less than 20 dB gain. The Analog aid was again about 3-4 dB below nominal gain, similar to the levels measured at 30 ms. The DigitalA aid had more gain at maximum amplitude than at 30 ms but was still 3-4 dB less than nominal gain. The DigitalB aid, once again, provided less gain than Analog and DigitalA hearing aids, but unlike the 30-ms measurement point, there was some measurable gain, albeit 12–15 dB less than nominal gain. There was no significant interaction between Input Level and Hearing Aid, so the results reported here are the analyses of the Main Effects.

Effect of Input Level: the 30 dB SPL input level resulted in significantly less gain than the 50 and 70 dB SPL input levels. 

Effect of Hearing Aid: DigitalB hearing aid provided significantly less gain than either the Analog or DigitalA hearing aids, and there was no significant difference between the gain provided by Analog and DigitalA. 

#### 3.1.3. 20-dB Gain Condition: Short Stimulus—Rise Time 

The rise time was the time taken to reach maximum amplitude (or, more precisely, the time to reach 90% of maximum amplitude). There was a clear trend for the two digital aids to take longer to reach maximum amplitude than the Analog aid. Rise time for the Analog aid was about 22 ms, similar to the 20-ms rise time of the input stimulus. Rise times for DigitalA ranged from 28 to 39 ms, and DigitalB showed a markedly longer rise times than DigitalA, approximately 45 ms.

Effect of Input Level: for the Analog and DigitalB hearing aids, rise time did not differ by input level (*p* > .1). For DigitalA, measured rise time differed by input level. The measured rise time was shortest for the 50 dB SPL input level (50 versus 70: *p* < .05), longer for the 70 dB SPL input level, and longer still for the 30 dB SPL input level (30 versus 70: *p* < .05).

Effect of Hearing Aid: at every input level, the rise time differed across hearing aids (*p* < .01), with the rise time being longest for the DigitalB hearing aid, shorter for the DigitalA hearing aid, and shortest for the Analog hearing aid. 

#### 3.1.4. 40-dB Gain Condition: Short Stimulus—Gain at 30-ms after Stimulus Onset

Mean gain values in Table 2 indicate that at the 30 ms measurement point, all hearing aids provided less than 40 dB gain. The Analog hearing aid provided 3–5 dB less than nominal gain, which was similar to the 20-dB gain condition. DigitalA provided 5–10 dB less than nominal gain, and DigitalB provided almost 20 dB less than the nominal 40 dB gain.

Effect of Input Level: unlike the 20-dB gain condition, where less gain was measured for the 30 dB SPL input level compared to the higher input levels, for the 40-dB gain condition, only DigitalA was measured to have less gain for the 30 than 50 dB input levels (*p* < .1). The Analog and DigitalB aids were measured to have the same amount of gain for both 30 and 50 dB SPL input levels (*p* > .1).

Effect of Hearing Aid: similar to the 20-dB gain condition, in the 40-dB gain condition, the DigitalB hearing aid provided significantly less gain than the DigitalA and Analog hearing aids at both input levels (*p* < .1). There was a nonsignificant trend for the DigitalA to provide less gain than the Analog hearing aid for the 30 dB SPL input (*p* = .11). The DigitalA and Analog hearing aids provided equivalent gain for the 50 dB SPL input (*p* > .1).

#### 3.1.5. 40-dB Gain Condition: Short Stimulus—Gain at Maximum Amplitude 

Mean gain values follow much the same pattern at maximum amplitude for the short stimulus as at 30 ms. Once again, all of the hearing aids provided less than 40 dB gain: Analog was again 3–5 dB below nominal gain, DigitalA was 4-5 dB below nominal gain, and DigitalB provided much less gain than both Analog and DigitalA hearing aids at about 12–14 dB below nominal gain. There was not a significant interaction between Input Level and Hearing Aid, so the results reported here are the analyses of the significant Main Effects. 

Effect of Input Level: the 30 dB SPL input level resulted in significantly less gain than the 50 dB SPL input level. 

Effect of Hearing Aid: DigitalB hearing aid provided significantly less gain than either the Analog or DigitalA hearing aids (*p* < .01), and there was no significant difference between the gain provided by Analog and DigitalA (*p* > .1). 

#### 3.1.6. 40-dB Gain Condition: Short Stimulus—Rise Time 

Again, there was a clear trend for the two digital aids to take longer to reach maximum amplitude than the analog aid, and the DigitalB aid took longer than DigitalA. The analog aid again mimicked the rise time of the input signal, with a measured rise time of about 22 ms. The DigitalA had a longer rise time, ranging from 22 to 34 ms. Again, DigitalB had a markedly longer rise time, taking about 41 ms to reach maximum amplitude.

Effect of Input Level: for the Analog aid, rise time did not differ between the two input levels (*p* > .1). For both the DigitalA and DigitalB hearing aids, measured rise time was longer for 30 dB SPL than the 50 dB SPL input level (*p* < .1).

Effect of Hearing Aid: at the 30 SPL input level, DigitalB had a longer rise time than both the DigitalA (*p* < .05) and Analog (*p* < .001) hearing aids. DigitalA had a longer rise time than Analog (*p* < .05). At the 50 dB SPL input level, DigitalB still had a longer rise time than both DigitalA (*p* < .001) and Analog (*p* < .001), but DigitalA and Analog had equivalent rise times (*p* > .1). 

### 3.2. Long Stimulus

**Figure 4:**
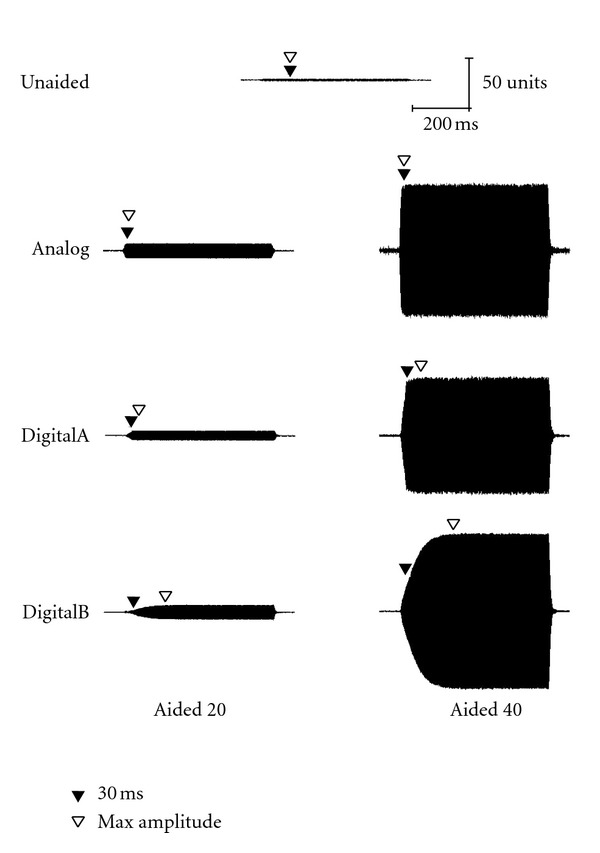
Waveform of the long stimulus presented at 30 dB SPL as measured in the ear canal of a single representative subject (subject 2).

**Figure 5:**
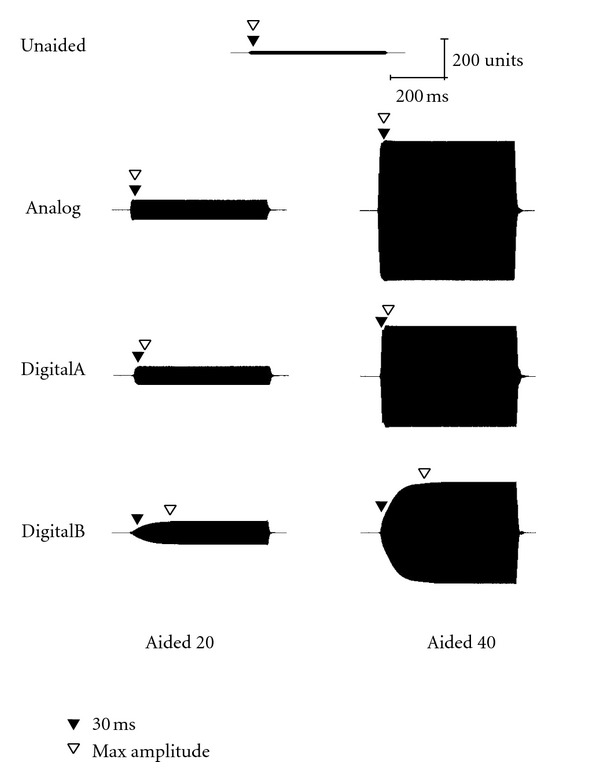
Waveform of the long stimulus presented at 50 dB SPL as measured in the ear canal of a single representative subject (subject 2).

**Figure 6:**
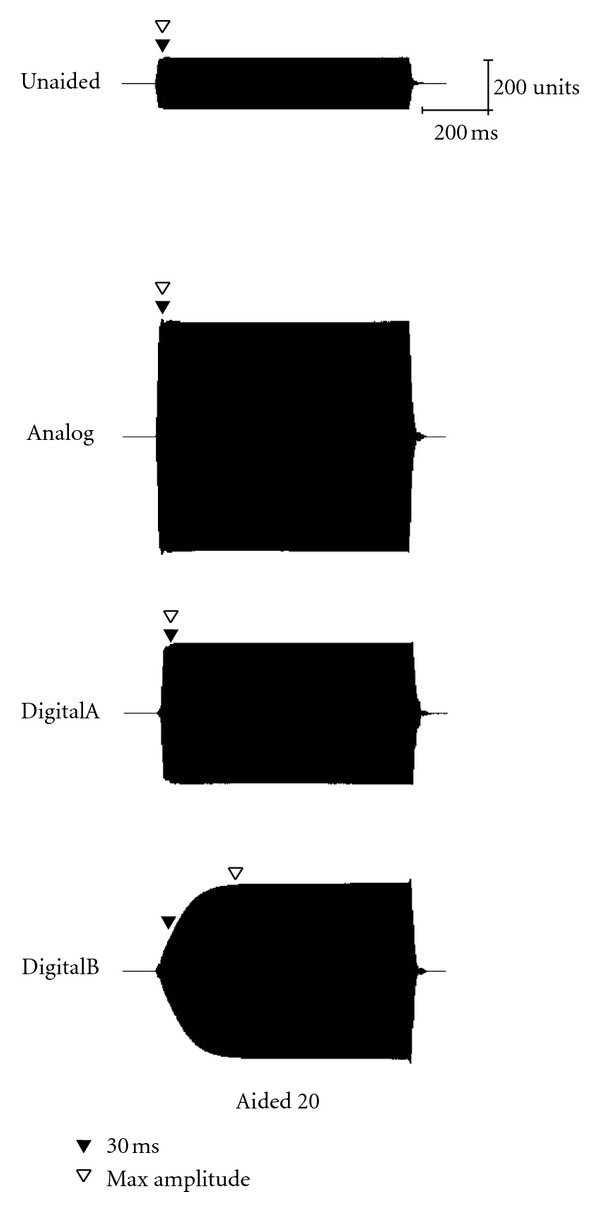
Waveform of the long stimulus presented at 70 dB SPL as measured in the ear canal of a single representative subject (subject 2).

Acoustic waveforms for the long stimulus (757-ms duration) in both unaided and aided conditions (Analog, DigitalA, and DigitalB) are presented for 30, 50, and 70 dB SPL input levels in Figures 4, 5, and 6, respectively. Both 20- and 40-dB gain settings are depicted where appropriate (e.g., 30 and 50 dB SPL) and 30-ms and maximum-amplitude measurement points are depicted by closed and open triangles, respectively. Once again, all figures in the following section illustrate the acoustic measures for a single representative subject (subject no. 2).

#### 3.2.1. 20-dB Gain Condition: Long Stimulus—Gain at 30 ms after Stimulus Onset 

Mean gain values in Table 2 indicate that at the 30-ms measurement point for the long stimulus, once again all of the hearing aids provided less than 20 dB gain, in a pattern similar to that found for the short stimulus. The analog aid was 3-4 dB below nominal gain across input levels; DigitalA was 3–7 dB below nominal gain, and DigitalB ranged from 4 dB gain down to 2 dB of attenuation.

Effect of Input Level: again, although all three hearing aids were set to provide linear amplification, in general the measured gain increased slightly as input level increased. For the Analog aid, equivalent gain was measured for the 30 and 50 dB SPL inputs (*p* > .1), and the gain measured for the 70 dB SPL input was greater than both the lower input levels (*p* < .1). For the DigitalA hearing aid, less gain was measured for the 30 compared with the 50 and 70 dB SPL input levels (*p* < .05) and equivalent gain for 50 and 70 dB SPL inputs (*p* > .1). Finally, the DigitalB hearing aid provided less gain for the 30 compared with the 50 (*p* < .05) and 70 dB SPL inputs (*p* < .01) and less gain for the 50 compared with the 70 dB SPL input (*p* < .1). 

Effect of Hearing Aid: the DigitalB hearing aid provided significantly less gain than both the DigitalA and Analog hearing aids at every input level (*p* < .01). The DigitalA and Analog hearing aids provided equivalent gain for all input levels (*p* > .1). 

#### 3.2.2. 20-dB Gain Condition: Long Stimulus—Gain at Maximum Amplitude

Mean gain values presented in Table 2 show that, when measured at the maximum amplitude for the long stimulus, once again all of the hearing aids provided less than 20 dB gain. The gain values were similar to the maximum gain values obtained with the short stimulus, with the notable exception of DigitalB aid, which now measured only 3–7 dB below nominal gain. 

Effect of Input Level: for all three hearing aids, increasing amounts of gain were measured with increases in input level (*p* < .1). 

Effect of Hearing Aid: at each input level, there was no difference among hearing aids in the amount of gain measured (*p* > .1).

#### 3.2.3. 20-dB Gain Condition: Long Stimulus—Rise Time

Again, the figures and Table 2 show a clear trend for the two digital aids to take longer to reach maximum amplitude than the analog aid, with the DigitalB aid showing a markedly longer rise time than DigitalA. Rise time for the Analog aid was about 12 ms, only slightly longer than the 7.5-ms rise time of the stimulus. Rise time for DigitalA ranged from 23 to 40 ms, and DigitalB had a much longer rise time of about 140–150 ms.

Effect of Input Level: for DigitalB, the rise time measured for 30 dB was slightly shorter than the rise time measured for the 50 dB SPL input level (*p* < .05). For DigitalA, the rise time measured for 30 was longer than that measured for 50 and 70 dB SPL input levels (*p* < .1). For Analog, rise time was equivalent across input levels.

Effect of Hearing Aid: at every input level, the rise time differed across hearing aids (*p* < .01), with the rise time being longest for the DigitalB hearing aid, shorter for the DigitalA hearing aid, and shortest for the Analog hearing aid. 

#### 3.2.4. 40-dB Gain Condition: Long Stimulus—Gain at 30-ms after Stimulus Onset 

Mean gain values presented in Table 2 indicate that, similar to the 20-dB gain setting at 30 ms, all of the hearing aids provided less than 40 dB gain. DigitalB provided less gain than either the Analog or DigitalA hearing aids. The Analog aid provided about 5 dB less than nominal gain, similar to the 20-dB gain condition. DigitalA provided about 4–6 dB less than nominal gain, similar to the 20-dB gain condition, and DigitalB provided about 14–16 dB less than the nominal 40-dB gain.

Effect of Input Level: the DigitalB hearing aid provided less gain for the 30 than 50 dB input levels (*p* < .1). Both the DigitalA and Analog hearing aids provided equivalent gain across the two input levels (*p* > .1). 

Effect of Hearing Aid: once again the DigitalB hearing aid provided significantly less gain than both other hearing aids at both input levels (*p* < .01), and there was no significant difference in gain provided by Analog and DigitalA at either input level (*p* > .1). 

#### 3.2.5. 40-dB Gain Condition: Long Stimulus—Gain at Maximum Amplitude

Mean gain data indicate that, at maximum amplitude for the long stimulus, all three hearing aids provided gain that was within 3–5 dB of the nominal 40 dB gain for all input levels. There were no significant effects of Hearing Aid or Input Level in the ANOVA.

#### 3.2.6. 40-dB Gain Condition: Long Stimulus—Rise Time

Again, there was a clear trend for the two digital aids to take longer to reach maximum amplitude than the analog aid, and the DigitalB aid took longer than DigitalA. The Analog aid was measured to have a rise time of about 12 ms, DigitalA to have a rise time of 19–43 ms, and DigitalB to have the longest rise time at 145–155 ms.

Effect of Input Level: both DigitalA and DigitalB had longer measured rise times for the 30 than 50 dB SPL input level (*p* < .1). Analog had equivalent rise times at the two input levels (*p* > .1).

Effect of Hearing Aid: for both input levels, DigitalB had a longer rise time than both DigitalA and Analog, and DigitalA had a longer rise time than Analog (*p* < .05). 

## 4. Discussion

All three hearing aids provided 20 and 40 dB of insertion gain at mid- and high-level inputs for all subjects when measured with a conventional hearing aid test system (Fonix 7000). When measuring the hearing aids with the stimuli used for the SCP measures, however, all of the hearing aids were measured to have less gain, particularly the two digital aids. The amount of gain reduction differed between the two digital hearing aids, with DigitalB showing much less gain than DigitalA in almost every condition. The two digital hearing aids, DigitalB in particular, reached their maximum gain well past 30 ms after stimulus onset. When the hearing aids were measured with a long stimulus at their maximum gain, there were no longer any differences among hearing aids in the amount of gain measured, but all three hearing aids demonstrated about 3–5 dB less gain than the gain measured with conventional REIG procedures.

The first main finding was that the maximum gain of all of the hearing aids was about 3–5 dB less than nominal gain even when a long stimulus was used. Although the conventional and SCP acoustic measures were made with different sets of equipment, different signals, and in different rooms, these effects cannot account for reductions in measured gain. Both acoustic measures (i.e., standard clinical measures and measures of the SCP stimuli) were insertion gain; thus, gain was always calculated as the aided response minus the unaided response. Additionally, the stimulus for both measures was a 1000-Hz pure tone, at the same input levels. Insertion gain is robust to differences in probe-tube insertion depth, measurement bandwidth, and small changes in room acoustics, particularly if the hearing aid has linear processing [26–28], which was the case for all three hearing aids in this study. Care was taken to ensure that head movement did not lead to substantial changes in the soundfield during SCP testing, which was calibrated with a substitution method rather than the on-line corrections of the commercial hearing aid test system. Because this difference was found for both the analog and digital hearing aids, it is not due to the type of processing (analog versus digital) or programming. 

It was a consistent finding that less gain was provided by digital hearing aids for the 30 dB SPL input level; this is likely due to the low-level expansion in both hearing aids, which could not be changed or disabled in the programming. Gain for a 30 dB SPL input was not measured with the standard hearing aid test system, as signals that low are not provided. However, reduced gain for low-level inputs cannot explain why nominal gain was not achieved even at higher input levels.

The second main finding was that both digital hearing aids altered the rise times of the stimuli such that there was a significant delay for both hearing aids to reach their maximum gain, and the amount of delay differed significantly between the two digital aids. This might be expected because of the commonly reported delays associated with digital processing [1, 29–35]. However, processing delays cannot account for the altered rise times measured for the SCP stimuli in this study for two reasons: first, processing delay was removed from the calculation of rise time by determining the 0-ms point as the time at which periodicity was first noted in the recording, rather than the time of signal presentation; second, even if this method did not fully remove the effects of processing delay, the results are inconsistent with the electroacoustic measures of delay. Electroacoustic measures of delay conducted on the Fonix 7000 system indicated that both digital hearing aids had longer delays (6.8 ms and 2.3 ms for DigitalA and DigitalB, resp.) compared with the Analog hearing aid (0.4 ms). Recall that the stimulus rise time was 20 ms for the short stimulus, so maximum amplitude would not be expected until 20 ms. Any processing delays could cause the maximum amplitude to be reached later than 20 ms. DigitalA did reach its maximum amplitude by 28 ms in some conditions, which is close to what would be expected if the electroacoustic measure of delay (6.8 ms) was added to the 20 ms stimulus rise time. In some conditions, however, DigitalA did not reach its maximum amplitude until 39 ms, beyond what could be explained by processing delay. Perhaps a stronger argument against the conventional measure of processing delay as an explanation for these results is that DigitalB, measured with the Fonix system to have only 2.3 ms processing delay, had the longest measured rise times, of 40 ms for the short stimulus and 150 ms for the long stimulus. Note the difference in measured rise times between short and long stimuli is due to the characteristics of the input signal; at 40 ms, the short stimulus was beyond its plateau and beginning to decrease in amplitude. 

These changes in altered rise time are also unlikely to be due to hearing aid processing parameters. All of the hearing aids were set (and subsequently verified) to linear processing. Any compression processing, had it remained on, would be expected to have the opposite effect as found here; that is, compression would be associated with faster rise times than measured here due to the overshoot that results from compression attack time [36, 37]. All other features were disabled, but again, features such as noise reduction or feedback reduction would demonstrate the opposite effect to the one measured in this study; that is, those features would be expected to show a gradual decrease in gain for the nonspeech pure tone [38, 39]. Thus, it is not immediately apparent what could account for the two main acoustic findings of this study. Because of the unknown and somewhat random differences between the two digital hearing aids, it is clear that the stimulus used for testing aided SCP responses must be carefully evaluated with acoustic measures across a range of hearing aid types and ultimately with typical processing features enabled. 

It is worth noting that the issues identified in this study are unlikely to be problematic only when using tonal stimuli, even though tonal stimuli have proven to be troublesome for measuring digital hearing aid processing [16, 39, 40]. Tremblay et al. [6] used speech stimuli to examine the effects of amplification on SCP responses and found that even providing 12–26 dB of gain had no effect on N1-P2 amplitude. Detailed acoustic analysis of their stimuli was not provided, but the lack of an amplification effect for speech stimuli suggests that the hearing aid processing altered their speech stimuli in such a way that affected the SCP measurements.

### 4.1. How Well Did These Acoustic Measures Predict the SCP Responses?

In our previous study [11], we demonstrated that the SCP responses measured for hearing-aid processed signals did not have the expected increases in amplitude and decreases in latency that would be predicted from 20 or 40 dB of gain added by the hearing aids. In the acoustic measures of the current study, we demonstrated that (a) the hearing aids failed to achieve 20 or 40 dB of gain when measured with the SCP stimuli and (b) the digital hearing aids, in particular, reached their maximum gain much later than 30 ms after stimulus onset. The acoustic waveforms show that shape varies across hearing aids for both short- and long-duration stimuli, particularly the onset, which is reflected in the measured rise times. The maximum gain is reached more gradually in DigitalB. 

**Figure 7:**
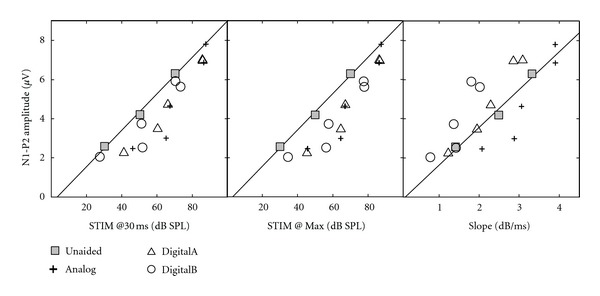
Group mean SCP amplitude from Marynewich et al. [11] as a function of three acoustic parameters: stimulus level at 30 ms (left panel), maximum stimulus level (middle panel), and onset slope (right panel) for unaided and 3 hearing-aid conditions. The fit line is the linear regression for the unaided condition.

To determine whether any of the measured acoustic parameters could predict the SCP response, we conducted an analysis of the group mean data from both studies. Although a thorough answer to this question would require a larger-scale parametric investigation of the relationship between acoustic variables and the SCP responses, some initial exploration of the findings can be instructive. Specifically, we used the group-mean SCP amplitude from Marynewich et al. [11] and developed a model of the relationship between acoustic measures and N1-P2 amplitude using the unaided responses. That is, we calculated the linear regression between N1-P2 amplitude and each of the three acoustic parameters: stimulus level at 30 ms, maximum amplitude, and slope of onset. See Figure 7 for stimulus level at 30 ms (left panel), maximum stimulus level (middle panel), and onset slope (right panel), where the data for the unaided condition are shown as shaded squares and the fit line is the linear regression for the unaided condition. The data for the aided conditions are also plotted on each panel. To determine how well each acoustic parameter predicted the N1-P2 amplitude in the aided conditions, we calculated the absolute value of the error between the actual mean aided SCP amplitudes and the SCP amplitudes predicted from the unaided data. As a rough estimate of overall how well each acoustic parameter could explain the data observed, we averaged the error across all input, gain, and hearing aid conditions.

The results of this analysis showed that both stimulus amplitude at 30 ms (average error: 1.08 *μ*V) and slope of onset (average error: 1.08 *μ*V) predicted the SCP amplitude equally well. The greatest amount of error was seen for maximum stimulus amplitude (average error: 1.35 *μ*V). The analysis is limited because it was performed on group mean data for two different groups of subjects. However, it is likely that the group analysis is representative of individual analysis for several reasons: four participants were in both studies; the hearing aids were set for individual ears; all participants had normal hearing; the acoustic measures generally had low variability. We can cautiously interpret this analysis to mean that the effect of hearing-aid processing on the onset characteristics of the stimulus had a greater influence on SCP amplitude than did the effect of hearing-aid processing on maximum stimulus amplitude. Recall that gain at 30 ms was generally much lower than the nominal gain, and particularly for DigitalB often measured close to 0 dB gain for the 20-dB gain condition. Thus, if the SCP was responding to the first 30 ms after stimulus onset, it is not surprising that primarily there was often little to no difference between aided and unaided acoustic measures, especially for the digital aids. These results are consistent with the view that approximately the first 30 ms of stimulus onset largely determines SCP N1 presence and amplitude [4, 18–21]. However, this interpretation cannot explain why even the Analog hearing aid only showed significant increases in SCP amplitude for the higher input levels.

### 4.2. Stimulus Level versus SNR

**Figure 8:**
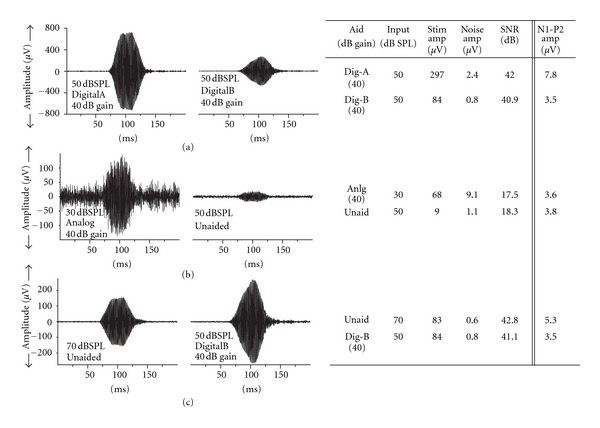
Three examples measured for one participant to show the relationship between stimulus level, SNR, and SCP amplitude. (a): stimulus level predicts SCP amplitude; (b): SNR predicts SCP amplitude; (c): neither stimulus level nor SNR predict SCP amplitude.

Because Billings et al. [2, 3, 24] hypothesized that their SCP results were due to the SNR in the aided condition, we conducted a brief analysis of SNR for one of our participants. We chose to do the analysis for one participant who had participated in both the current study and in our previous study [11]. For this ad hoc analysis, we measured signal and noise levels on single-trial recordings rather than averages. Noise was the RMS level in a 1/3rd-octave band centred at 1000 Hz measured over a 70-ms period prior to stimulus onset. For each stimulus condition, measures of the noise levels were made for three separate samples and averaged. Stimulus level was the amplitude at 30 ms, as reported in Section 3. From these calculations, we found that SNR might explain some of the results in the SCP response, but not all of them; generally there was very little noise in the acoustic recordings, even in the aided condition. Three examples have been chosen to illustrate the relationship between SNR, stimulus level, and N1-P2 amplitude; these are shown in Figure 8.

Panel (a) of Figure 8 shows an example where SCP N1-P2 amplitude seems best related to the stimulus amplitude at 30 ms. The first column of Panel (a) shows a single-trial waveform for DigitalA aid at a 50 dB SPL input level with 40 dB gain. The second column shows a single-trial waveform for DigitalB at 50 dB SPL with 40 dB gain. The table to the right provides the relevant data points for input level, stimulus level at 30 ms, noise level, SNR, and SCP N1-P2 amplitude. In this example, although the SNRs are similar for the two conditions, SCP amplitude differs in the same way as the stimulus level changes. 

Panel (b) of Figure 8 is an example that is in agreement with Billings et al. [2, 3, 24], where the SCP response seems to be related to SNR. Comparing Unaided for a 50 dB SPL input level to Analog 30 dB SPL input plus 40 dB gain, the SCP N1-P2 amplitudes are almost identical. In this case, the stimulus levels are very different, but the SNRs are similar and both could be considered good. In this case, the good SNR might be the predictor of the SCP response rather than the stimulus level.

Finally, in Panel (c), there is an example where neither stimulus amplitude at 30 ms nor SNR seems to be good predictors of the SCP response. In this example, comparing 70 dB SPL unaided to DigitalB 50 dB SPL input +40 dB gain, these measures have similar stimulus levels and similar (good) SNRs, yet the SCP response for DigitalB is much lower than unaided. In this case, neither stimulus level nor SNR can explain the different SCP responses observed.

Altogether, this set of examples shows that SCP amplitudes in our data set may be accounted for by changes to rise time in some conditions, SNR in some conditions, and some other, as yet unidentified, acoustic parameter in other conditions. 

## 5. Conclusions

In the present study, we attempted to determine why several recent studies, including our own study [11], have been unsuccessful in demonstrating a significant amplification effect on SCP measures. We reduced sources of unknown variability as much as possible by using hearing aids with linear processing and all features disabled. The acoustic measures of the amplified SCP stimuli showed that (a) the hearing aids all provided less than expected gain for these stimuli and (b) the digital hearing aids took longer to reach their maximum gain than the analog hearing aid. The acoustic measures of stimulus level at 30 ms and onset slope were predictive of SCP response amplitude, but it is likely that additional acoustic characteristics, not measured in this study, contributed to SCP response.

In light of findings from the present study, it is likely that prior studies using speech or tonal stimuli [2, 4–9, 41] were measuring SCPs to stimuli that were substantially altered by the hearing aid in a way that was not quantified. For instance, different speech stimuli may not result in distinct neural response patterns if the hearing-aid processed stimuli are altered in such a way that they are acoustically very similar. Likewise, the same stimulus may be altered in different ways by the same hearing aid, as was the case in the current studies. 

Prior studies on hearing-aid processed click and brief-tone stimuli (typically used for ABR testing) reported considerable variability among hearing aids in terms of gain provided to onset and steady-state portions of transient stimuli [12, 13], thus, these stimuli were determined to be too short for measures of hearing-aid processing. The longer-duration stimuli used for SCP testing were thought to be long enough to overcome this problem [1]; however, findings from the current studies indicate that a tonal stimulus with parameters appropriately set to elicit large unaided N1 amplitudes is still too brief to measure hearing aid gain, particularly those with digital processing, despite the hearing aids being set to provide linear gain with all advanced processing features disabled. 

The less-than-expected measureable gain resulting from hearing-aid processing for SCP stimuli suggests that SCP stimuli do not provide appropriate measures of hearing aid gain. Our acoustic analysis shows that changes to rise time, particularly in ways that affect stimulus amplitude at 30 ms, may explain our previous findings [11]. However, we cannot rule out SNR, or even another acoustic parameter, as a potential contributor to the SCP measures of Marynewich et al. [11]. As a result of these unknown factors, more research concerning aided-SCP testing is needed for clinical application of this technique, and any results must be interpreted very cautiously if used within the hearing-aid fitting process. As has been noted by others, the lack of an SCP response does not ensure that the stimulus is inaudible [42–44]; similarly, a “present” aided SCP does not ensure that the stimuli are sufficiently audible [10]. 

Although our studies included only participants with normal hearing, the acoustic alterations of the stimuli that we measured are independent of hearing status. The concerns raised by these studies indicate that much is unknown about the application of SCP measures in hearing aid fitting. Future research might involve (i) additional hearing aid measures to determine the source of alteration to rise time and (b) parametric study of the relationship between the stimulus acoustic measures and the SCP responses. Future research might also explore different/more-appropriate SCP stimuli or presentation paradigms for hearing aid measures to determine under what conditions aided SCP measures would be valid.

## Figures and Tables

**Table 1:** Real-ear insertion gain (REIG) (dB) measured with a hearing aid test system for 20 and 40 dB gain settings.

Gain setting (dB)	20		40
Input level (dB SPL)	50	70	50
Subject	Analog		

1	20.40	20.70	39.90
2	19.90	20.00	39.80
3	20.00	19.70	39.40
4	20.00	20.70	39.70
14	20.50	19.30	39.90

Mean	20.16	20.08	39.74
SD	0.27	0.62	0.21

Subject	Digital A		

1	20.30	19.80	40.40
2	20.30	20.10	39.60
3	19.50	19.70	39.90
4	20.10	20.30	40.30
14	20.10	19.40	40.40

Mean	20.06	19.86	40.12
SD	0.33	0.35	0.36

Subject	Digital B		

1	20.10	20.00	40.10
2	20.10	20.60	40.10
3	19.80	19.70	40.40
4	20.00	20.10	40.10
14	20.00	20.20	39.90

Mean	20.00	20.12	40.12
SD	0.12	0.33	0.18

**Table 2:** Mean (*n* = 5) and standard deviation for gain measured at (i) 30 ms and (ii) maximum amplitude, and rise time (measured at 90% of maximum amplitude) for the cortical stimuli.

Nominal gain (dB) Input (dB SPL)	Short stimulus (60 ms)						Long stimulus (757 ms)				
	20			40			20			40	
	30	50	70	30	50		30	50	70	30	50
Analog											
30 ms (dB)	15.9 ± 1.6	17.0 ± 0.9	17.3 ± 1.0	34.9 ± 2.2	36.2 ± 1.8		16.0 ± 0.7	16.7 ± 0.6	17.4 ± 0.7	35.5 ± 0.5	35.5 ± 1.4
Max (dB)	15.7 ± 1.1	16.9 ± 1.0	17.4 ± 0.1	34.5 ± 1.9	36.2 ± 2.0		15.2 ± 1.3	16.6 ± 0.7	17.3 ± 0.8	34.7 ± 1.9	35.3 ± 1.5
Rise time (ms)	22.2 ± 1.9	21.9 ± 1.4	22.4 ± 1.7	22.5 ± 1.4	22.1 ± 1.9		12.5 ± 3.2	11.6 ± 3.2	12.8 ± 2.8	11.9 ± 1.9	12.3 ± 2.6
DigitalA											
30 ms (dB)	10.9 ± 4.5	15.8 ± 3.1	15.6 ± 3.1	30.1 ± 6.1	35.5 ± 3.5		13.1 ± 3.3	17.0 ± 2.0	16.8 ± 2.4	33.8 ± 3.2	36.1 ± 1.5
Max (dB)	15.4 ± 3.2	17.1 ± 2.7	16.7 ± 2.6	34.5 ± 4.3	36.3 ± 2.6		15.7 ± 2.4	17.8 ± 1.6	17.1 ± 2.6	36.9 ± 1.9	36.4 ± 1.3
Rise time (ms)	38.6 ± 3.7	27.9 ± 3.3	30.3 ± 3.1	33.8 ± 6.6	22.6 ± 2.3		39.4 ± 7.2	25.7 ± 7.4	23.4 ± 6.6	43.3 ± 14.7	18.8 ± 5.5
DigitalB											
30 ms (dB)	−2.7 ± 2.1	0.9 ± 2.9	0.2 ± 2.3	21.5 ± 3.4	23.1 ± 2.3		−2.0 ± 3.2	1.4 ± 5.3	4.2 ± 3.9	23.1 ± 2.2	26.1 ± 2.6
Max (dB)	4.5 ± 3.8	7.6 ± 2.3	7.5 ± 1.1	26.2 ± 2.2	27.8 ± 1.6		13.8 ± 4.1	15.8 ± 3.1	17.4 ± 2.2	35.4 ± 1.8	37.0 ± 1.8
Rise time (ms)	45.5 ± 2.7	44.2 ± 1.8	45.2 ± 1.5	41.6 ± 2.4	40.9 ± 2.5		141.5 ± 8.9	150.8 ± 4.5	149.6 ± 8.4	154.5 ± 3.3	145.6 ± 3.2

**Table 3:** ANOVA results for all measures of the short stimulus. Shown are *p* values of the ANOVA and of the simple main effects, where appropriate.

	20 dB gain			40 dB gain		
	Amp 30 ms	Amp Max	Rise time	Amp 30 ms	Amp Max	Rise time
Main effects						
Hearing aid (HA)	***p* < .001**	***p* < .001**	***p* < .001**	***p* < .001**	***p* < .001**	***p* < .001**
Input level (LVL)	***p* = .001**	***p* = .054**	***p* < .001**	***p* = .045**	***p* = .087**	***p* = .004**
HA × LVL	***p* = .03**	*p* = .11	***p* < .001**	***p* = .036**	*p* = .99	***p* < .001**
Simple main effects						
Effect of LVL						
For analog	***p* = .018**	∗	*p* = .744	*p* = .309	∗	*p* = .484
For DigitalA	***p* = .004**	∗	***p* = .001**	***p* = .028**	∗	***p* = .007**
For DigitalB	***p* = .048**	∗	*p* = .121	*p* = .194	∗	***p* = .045**
Effect of HA						
At 30 dB SPL	***p* < .001**	∗	***p* < .001**	***p* = .001**	∗	***p* < .001**
At 50 dB SPL	***p* < .001**	∗	***p* < .001**	***p* = .001**	∗	***p* < .001**
At 70 dB SPL	***p* < .001**	∗	***p* < .001**	—	—	—

Boldface indicates significance at *p* < .1. ∗: Indicates the analysis was not necessary.  —: Indicates no data collected for those conditions.

**Table 4:** ANOVA results for all measures of the long stimulus. Shown are *p* values of the ANOVA and of the simple main effects, where appropriate.

	20 dB gain			40 dB gain		
	Amp 30 ms	Amp max	Rise time	Amp 30 ms	Amp max	Rise time
Main effects						
Hearing aid (HA)	***p* < 0.001**	*p* = 0.656	***p* < 0.001**	***p* < 0.001**	*p* = 0.314	***p* < 0.001**
Input level (LVL)	***p* < 0.001**	***p* = 0.005**	*p* = 0.424	***p* = 0.027**	*p* = 0.298	***p* = 0.016**
HA × LVL	***p* = 0.031**	***p* = 0.09**	***p* < 0.001**	***p* = 0.091**	*p* = 0.107	***p* = 0.029**
Simple main effects						
Effect of LVL						
For analog	***p* = 0.023**	***p* = 0.009**	*p* = 0.647	*p* = 0.956		*p* = 0.429
For digitalA	***p* = 0.006**	***p* = 0.067**	***p* = 0.02**	*p* = 0.134		***p* = 0.030**
For digitalB	***p* = 0.002**	***p* = 0.003**	***p* = 0.07**	***p* = 0.015**		***p* = 0.007**
Effect of HA						
At 30 dB SPL	***p* < 0.001**	*p* = 0.457	***p* < 0.001**	***p* < 0.001**		***p* < 0.001**
At 50 dB SPL	***p* < 0.001**	*p* = 0.377	***p* < 0.001**	***p* < 0.001**		***p* < 0.001**
At 70 dB SPL	***p* < 0.001**	*p* = 0.97	***p* < 0.001**			

Boldface indicates significance at *p* < .1. ∗: Indicates the analysis was not necessary.  —: indicates no data collected for those conditions.
